# Prevalence and Age-Related Changes of Corneal Astigmatism in Patients Undergoing Cataract Surgery in Northern China

**DOI:** 10.1155/2020/6385098

**Published:** 2020-09-29

**Authors:** Zhizhong Wu, Caijuan Liu, Zhimin Chen

**Affiliations:** Department of Ophthalmology, Hebei Eye Hospital, Xingtai, Hebei, China

## Abstract

**Purpose:**

To examine the magnitude, orientation, and age-related changes of corneal astigmatism of the eyes before cataract surgery. *Setting*. Hebei Eye Hospital, Hebei, China.

**Design:**

A clinical-based retrospective study.

**Results:**

The study consisted of 5662 eyes of 5662 consecutive cataract surgery patients with a mean age of 68.26 ± 10.39 (mean ± standard deviation (SD)) years (range 40 to 97 years), and 59.86% of the patients were women. Mean corneal astigmatism was 0.98 ± 0.76 diopter (D) (range 0.00–9.61 D). Corneal astigmatism of 0.50–0.99 D was the most common range of values (30.08%), followed by 1.00–1.49 D (22.15%), ≤0.50 D (21.21%), and 1.50–1.99 D (10.28%). There was a strong U-shaped relation between corneal astigmatism and age (*p* for nonlinearity <.01). With the increase of age, the astigmatism axis gradually changes from with-the-rule (WTR) to against-the-rule (ATR). Moreover, in young patients with age below 65 years, WTR astigmatism was negatively correlated with age, while ATR was positively correlated with age (*r* = −0.11, *p*=.001; *r* = 0.10, *p*=.010, respectively). However, in the old patients with age above and equal to 65 years, all types of astigmatism were positively correlated with age.

**Conclusion:**

This study may provide valuable and practical information to surgeons when selecting the appropriate surgical method and toric intraocular lens (IOLs).

## 1. Introduction

Cataract is the leading cause of blindness worldwide, and it is one of the most frequently performed surgeries globally. Cataract surgery has also gradually become less invasive and more precise. Its goal has changed from improving visual acuity to providing optimal vision and minimizing postoperative spectacle dependence [1]. Corneal astigmatism is a major concern in modern cataract surgery, which provides a unique opportunity for the surgeon to address the issue of corneal astigmatism before surgery, in order to achieve the desired postoperative visual quality. The common techniques to treat corneal astigmatism during cataract surgery include incisions on the steep axis, peripheral corneal-relaxing incisions (PCRIs), toric IOLs, or their combinations [1–3]. Corneal astigmatism after cataract surgery forward continues to change [4]; therefore, the surgeon should properly overcorrect or undercorrect the corneal astigmatism, according to the long-term changes of corneal astigmatism [4, 5].

To date, previous studies reported the prevalence of preoperative astigmatism in different populations [4–16]. However, the cataract patients were mainly from a single hospital, and the results do not completely represent the data of the whole population in their regions. Therefore, more similar studies need to be conducted. Our goal was to examine the magnitude, orientation, and age-related changes of corneal astigmatism of the eyes before cataract surgery in northern Chinese patients.

## 2. Materials and Methods

### 2.1. Study Population

This is a retrospective study of 5662 consecutive patients who attended the cataract surgery in the Ophthalmology Department of the Hebei Eye Hospital between January 2018 and June 2019. The study adhered to the Declaration of Helsinki and was approved by the Hebei Eye Hospital Ethical Committee. Informed consent was obtained from the subjects. Patients with a history of corneal disease (including corneal ectasia and secondary irregular astigmatism) or intraocular surgery, ocular trauma, contact lens wear, irregular astigmatism, and corneal diseases that did not allow IOL Master measurement were excluded from the study.

### 2.2. Data Collection

The medical and systematic histories of the patients were reviewed. The patient underwent a comprehensive eye examination, including slit lamp biomicroscopy, applanation tonometry and fundus ophthalmoscopy through dilated pupils. The data points extracted for the study included age, sex, and the magnitude and orientation of corneal astigmatism. All reported measurements of corneal astigmatism refer to values obtained from the IOL Master 500 (Carl Zeiss Meditec, Jena, Germany, software version 5.4). Experienced technicians measured keratometry data with, at least, three automatic measurements performed in each eye.

Corneal astigmatism was designated as with-the-rule (WTR) when the axis of correcting minus cylinder was within 180 ± 30 degrees (the steep meridian of the cornea being within 90 ± 30 degrees in this case), against-the-rule (ATR) when the correcting minus cylinder axis was within 90 ± 30 degrees, and oblique if it was neither WTR nor ATR.

### 2.3. Statistical Analysis

Data were tested for normal distribution using the Kolmogorov–Smirnov test. Descriptive statistics are the mean ± standard deviation (SD) for normally variables and median values/interquartile range for nonnormal variables. Frequency and percentages were used for categorical variables. Comparisons among the age groups were performed using nonparametric tests for continuous variables and the *χ*^2^ test for categorical variables, respectively. The Bonferroni method was used for post hoc pairwise comparisons. Associations between corneal astigmatism and age were determined using Spearman's correlation analysis and nonlinear regression analysis. *p* values of <.05 were considered statistically significant. All statistical analyses were performed using IBM SPSS Statistics Version 25 and *R* Version 3.6.0. Graphs were generated with GraphPad Prism 8.

## 3. Results

### 3.1. Baseline Characteristics

**Figure 1:**
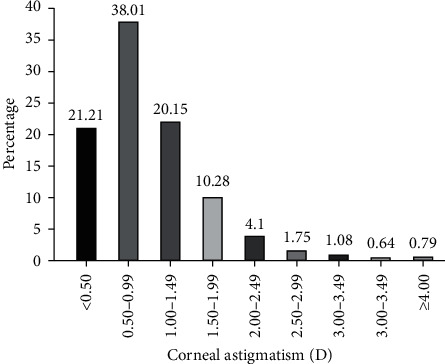
Distribution of corneal astigmatism (D).

A total of 5662 eyes of 5662 patients were included in the study, of which 3389 were females. The mean age of patients was 68.26 ± 10.39 years (range 40–97 years). Mean corneal astigmatism was 0.98 ± 0.76 diopter (D) (range 0–9.61 D) (Table 1). The frequency distribution of corneal astigmatism for all patients is shown in Figure 1. Corneal astigmatism of 0.50–0.99 D was the most common range of values (30.08%), followed by 1.00–1.49 D (22.15%), ≤0.50 D (21.21%), and 1.50–1.99 D (10.28%). The magnitude of corneal astigmatism was 2.00 D or above in 473 (8.35%) eyes. No significant deviation was found measured separately for men and women (*Z* = 1.16, *p*=.138).

### 3.2. Corneal Astigmatism in Different Age Groups

**Figure 2:**
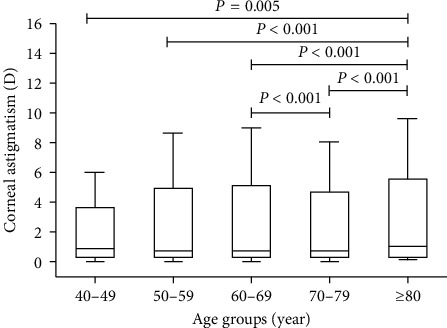
Corneal astigmatism in different age groups. Boxes show the interquartile range. Bold lines in boxes represent the median, and the whiskers represent extreme values.

In the comparison between the age groups, patients over 80 years of age had the highest level of corneal astigmatism of 1.00 (0.57–1.50) *p* < .005. The astigmatism values of each age group were 0.81 (0.50–1.25) for 40–49 years of age, 0.75 (0.48–1.19) for 50–59 years of age, 0.75 (0.50–1.12) for 60–69 years of age, and 0.75 (0.50–1.25) for 70–79 years of age (Figure 2).

### 3.3. Association of Corneal Astigmatism and Age

**Figure 3:**
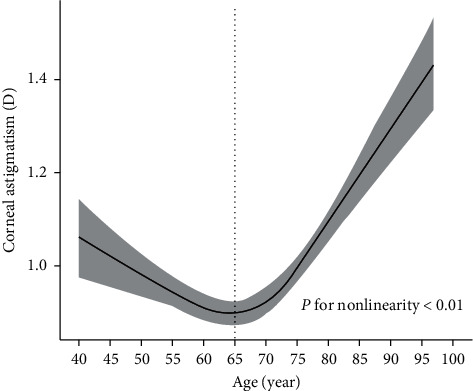
Association between corneal astigmatism and age. Corneal astigmatism is indicated by a solid line, and 95% confidence interval is indicated by the shaded area.

We used restricted cubic splines to the flexibly model and visualized the associations stratified by corneal astigmatism and age. Regarding the strong U-shaped relation between corneal astigmatism and age, the plot showed a substantial reduction of corneal astigmatism within the lower range of age, which reached the lowest corneal astigmatism around the age of 65 years and, then, increased thereafter (*p* for nonlinearity <.01) (Figure 3).

### 3.4. Distribution of the Astigmatic Axis in Different Age Groups

**Figure 4:**
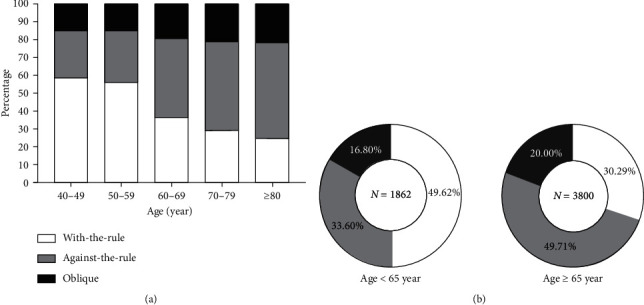
Relationship between corneal astigmatism axis and age. (a) Changes in corneal astigmatism axis with age. (b) Differences in the corneal astigmatism axis in age groups.

There was a shift in corneal astigmatism from WTR to ATR with advancing age, manifested as the proportion of ATR and oblique astigmatism increased, whereas the proportion of WTR astigmatism decreased as a function of age. We found a linear trend of the proportion of astigmatic axis types across the age groups (*p* < 0.5) (Figure 4(a)). In young patients with age below 65 years, 49.62%, 33.60%, and 16.80% of patients showed WTR, ATR, and oblique astigmatism, while in the old patients with age above and equal to 65 years, 30.29%, 49.71%, and 20.00% of patients showed WTR, ATR, and oblique astigmatism, respectively. There were statistical differences between the two age groups in the comparison of each type of astigmatic axis (*p* < 0.1) (Figure 4(b)).

### 3.5. Correlation between Corneal Astigmatism and Age in Three Types of Astigmatic Axis

When stratified by the two age groups and three types of astigmatic axis, the corneal astigmatism of young patients was negatively correlated with age in the WTR group, but positively correlated with age in the ATR group (*r* = −0.11, *p*=.001; *r* = 0.10, *p*=.010, respectively). There was no association between corneal astigmatism and age in the oblique group. However, the corneal astigmatism of old patients was positively correlated with age, regardless of the type of astigmatic axis (Table 2).

## 4. Discussion

This study evaluated the distribution of ocular biometric parameters and characteristics of corneal astigmatism in cataract surgery candidates in northern China. More and more surgeons begin to pay attention to the correction of corneal astigmatism in cataract patients. Moreover, the era of refractive cataract surgery has arrived, and refractive cataract is defined as the uncomplicated removal of cataract while minimizing postoperative spectacle dependence. Present focal point has been on the reduction or elimination of astigmatism rather than on minimizing postoperative spherical error [3]. Increasingly effective techniques are available for treating corneal astigmatism at the time of cataract surgery. A 3.2 mm clear corneal phacoemulsification incision results in surgically induced astigmatism of 0.5 *D* [17].Therefore, on-axis phacoemulsification is effective to treat the less than 1 D of corneal astigmatism. However, there are many restrictive factors such as wrist support, legroom beneath the operating table, operating microscope position, and other factors when choosing on-axis phacoemulsification [3]. Single or paired PCRIs are useful for correcting 1–1.5 D of regular corneal astigmatism when implanting spherical monofocal IOLs. However, the results can be unpredictable, and the procedure can be complicated by perforation, pain, placement on an incorrect axis, overcorrection, wound gape, and infection [18]. Toric IOLs correct moderate to severe corneal astigmatism, and hey can correct up to 8 D of astigmatism [19].Moreover, toric IOLs provided better uncorrected distance visual acuity, greater spectacle independence, and lower amounts of residual astigmatism than nontoric IOLs even when relaxing incisions were used [19–21].

Previous studies have shown that the proportion of corneal astigmatism of more than 1 D is between 41.3% and 67.39% [11, 14, 22, 23]. In our study, 59.22% of eyes had corneal astigmatism of 1.00 D or more, which was the highest compared with other studies on the Chinese population [10, 11, 24, 25].This is probably due to a regional difference in the populations, as well as different environmental factors. More and more researches show that toric IOLs are used as first-line measures for correcting corneal astigmatism of >1.5 D at the time of phacoemulsification [2, 3, 19]. This means that, in our study, 18.63% of patients could be associated with favorable results in corneal astigmatism correction with the use of toric IOLs, which is similar to the percentage in studies by Chen et al. [11] (18.49%) and De Bernardo M et al. [23] (17.57%).

The mean corneal astigmatism in this cohort was 0.98 ± 0.76 (range 0.00 to 9.61 D), which is comparable to 1.01 ± 0.69 by Chen et al. [11] and 0.98 ± 0.78 as reported by Hoffmann PC [13], whereas other studies in China have reported higher mean corneal astigmatism: 1.09 ± 0.77 by Yu et al. [24] and 1.15 ± 0.84 by Yuan et al. [25]. This is probably attributed to a regional difference in the populations, as well as the use of different inspection equipment. More and more studies have begun to pay attention to the relationship between age and corneal astigmatism. Similar findings showed that the magnitude of corneal astigmatism increased with increasing age [6, 25]. In current study, interestingly, we found a stronger U-shaped association between corneal astigmatism and age (Figure 3), which was not reported in most of the previous studies. Spearman's correlation analysis (Table 2) shows that the magnitude of corneal astigmatism decreased with increasing age in middle-aged cataract patients (<65 years of age) and increased in older patients (≥65 years of age). Therefore, because of this nonlinear trend, astigmatism correction is difficult in younger patients (<65 years of age).

We also found that there was a positive correlation in the prevalence of the ATR astigmatism with age (*p* < .01), which was consistent with conclusions of most previous articles [4–13, 22–26]. Moreover, similar trends occurred in the prevalence of the oblique astigmatism with age in current study (*p* < .01). In contrast, as in other published articles [5, 7, 8, 24, 25], the prevalence of WTR astigmatism decreased with increasing age (*p* < .01). The shift might be induced by possibly changes in corneal structure [27] and eyelid morphology and power [28]. Cataract surgery did not change these trends [29]. Therefore, these trends emphasize the need for special attention in long-term astigmatism treatment. With regard to correcting WTR astigmatism, the middle-aged patients should be considered that if corrected fully at the time of surgery, then there is the risk of shifting to ATR astigmatism in the later years [9]. Moreover, Table 2 shows the magnitude of ATR corneal astigmatism positively correlated in middle-aged and older patients (*r* = 0.10, *p*=.010; *r* = 0.16, *p* < .001, respectively). Previous studies found that ATR corneal astigmatism increased with age even after sutureless cataract surgery, and this change was similar to the aging process of the normal cornea [29, 30]. We suggest that cataract surgeons should consider treating ATR corneal astigmatism more aggressively with the aim to fully correct [5, 7, 8, 24] or overcorrect in long-term astigmatism treatment because if not, it would subsequently deteriorate, especially in the middle-aged cataract population.

This study has some limitations. First, cases included in the study in our hospital were not large enough to represent the whole population in China. Second, the effect of the posterior corneal surface was not taken into consideration in the current data. Ignoring the posterior surface astigmatism in the calculation of toric IOLs can cause more correction for the eyes that have with-the-rule astigmatism or undercorrection for the eyes that have against-the-rule astigmatism [31]. Moreover, the posterior corneal surface showed ATR astigmatism regardless of the age [32], and it will compensate the anterior corneal astigmatism in the younger population and increases the total astigmatism in the older population [33]. Measurement of the posterior corneal astigmatism would have to be performed as part of normal preoperative assessment of patients undergoing refractive cataract surgery.

In conclusion, the current study found a corneal astigmatism of 1.50 D or more in a significant number of eyes (18.63%), which could benefit from toric IOL implantation. Considering the trend of magnitude, orientation, and age-related changes of corneal astigmatism, ATR corneal astigmatism should be considered more aggressively with the aim to fully correct or overcorrect in long-term astigmatism treatment. Although the majority of our study population came from patients in our hospital, these data will not only expand the database of corneal astigmatism in the Chinese population but also provide valuable and practical information to surgeons when selecting the appropriate surgical method and toric IOLs.

## Figures and Tables

**Table 1:** Demographic characteristics and clinical findings of the study population.

Characteristic	Value
Eyes/patients (*n*)	5662/5662

Age (years)	
Mean (years)	68.26 ± 10.39
Range of age (years)	40 to 97
Female, no. (%)	3389 (59.86)

Corneal astigmatism (D)	
Mean corneal astigmatism (D)	0.98 ± 0.76
Range of corneal astigmatism (D)	0.00 to 9.61

Axis of astigmatism, no. (%)	
With-the-rule	2075 (36.65)
Against-the-rule	2514 (44.40)
Oblique	1073 (18.95)

Data are presented as numbers and percentages, means and standard deviations (SD). D = diopter.

**Table 2:** Spearman's correlation analysis of corneal astigmatism and age in three types of astigmatism axis.

Corneal astigmatism	Total		Age <65 years		Age ≥65 years	
	*r*	*p* value	*r*	*p* value	*r*	*p* value
With-the-rule astigmatism	−0.002	0.920	−0.105	0.001	0.094	0.001
Against-the-rule astigmatism	0.179	<0.001	0.102	0.010	0.166	<0.001
Oblique astigmatism	0.118	<0.001	−0.058	0.307	0.154	<0.001

## Data Availability

Data used for the analysis are available from the corresponding author upon reasonable request.
